# A Rare Case of Severe Cold Autoimmune Hemolytic Anemia in a Hemochromatosis Carrier

**DOI:** 10.1155/crh/7070088

**Published:** 2026-02-26

**Authors:** Justin H. Franco, Priya K. Jindal, Sarah Jaggernauth, Feehaan Sultan, Anu Garg

**Affiliations:** ^1^ Department of Internal Medicine, University of Toledo College of Medicine and Life Sciences, Toledo, Ohio, 43606, USA, utoledo.edu

**Keywords:** anemia, autoimmune, cAIHA, cirrhosis, hemochromatosis

## Abstract

Cold autoimmune hemolytic anemia (cAIHA) is a rare form of anemia characterized by antibody‐mediated red blood cell destruction at low temperatures (i.e., below 98.6°F or 37°C). Patients with severe cAIHA exhibit hemoglobin levels under 8 g/dL. Affected patients present with hepatosplenomegaly, hypotension, tachycardia, and an increased risk of death with a 5‐year survival rate of 63.5%. Treatment with immunosuppressants and lifestyle modifications can improve cAIHA symptoms. However, the presence of a concurrent disease process can worsen clinical outcomes. In our report, we discuss the clinical management of acute decompensated cirrhosis secondary to heterozygous hemochromatosis complicated by cAIHA. Hemochromatosis is characterized by increased iron deposition that results in organ dysfunction. On admission, the patient presented with anemia, weight loss, and ascites. Although heterozygous hemochromatosis is typically asymptomatic, the presence of cAIHA appears to have contributed to the patient’s liver dysfunction. Requiring different treatment regimens, the case underscores the difficulty of managing multiple rare hematological conditions.

## 1. Introduction

Autoimmune hemolytic anemia (AIHA) is a rare type of anemia that exhibits an incidence of 1.4–6.6 per 100,000 people/year in the United States [1–3]. The disease results from autoantibodies that target red blood cells (RBCs), thus leading to hemolysis and low hemoglobin (Hgb) [3, 4]. AIHA is classified as either warm, cold, or mixed depending on the responsible antibody type [5]. In cold AIHA (cAIHA), IgM autoantibodies cause hemolysis to occur at low temperatures (i.e., below 98.6°F or 37°C) [5–7].

Patients with cAIHA present with hepatosplenomegaly, hypotension, tachycardia, and acrocyanosis [6–8]. Although most patients will exhibit mild to moderate anemia (i.e., Hgb ≥ 8 g/dL), 27% of patients will experience severe hemolysis (i.e., Hgb < 8 g/dL) [9, 10]. This translates to 1‐year and 5‐year survival rates of 85.8% and 63.5%, respectively [11]. Current treatments rely on B‐cell directed therapies (e.g., rituximab) and complement inhibitors (e.g., sutimlimab and eculizumab) [12].

Underlying conditions can worsen cAIHA, such as hemochromatosis. In hemochromatosis, there is excessive iron deposition that leads to organ dysfunction [13, 14]. Affected patients present with skin hyperpigmentation, cirrhosis, and diabetes mellitus [13, 14]. Our case represents a unique instance of heterozygous hemochromatosis that is complicated by cAIHA.

## 2. Case Report

The patient is a 48‐year‐old female with past medical history significant for obesity (body mass index of 41.9 kg/m^2^), tobacco use (16.5 pack years), obstructive jaundice (status post endoscopic retrograde cholangiopancreatography and cholecystectomy), alcohol use disorder (sober since 1999), cAIHA, and heterozygous hemochromatosis. In January 2023, she was diagnosed with cAIHA after presenting with fatigue, dyspnea, and tachycardia. Lab work was significant for anemia (Hgb: 6.8 g/dL), reticulocytosis (11.8%), and reduced haptoglobin (< 30 mg/dL). Infection was ruled out by negative cultures (e.g., blood and urine) and unremarkable imaging. Likewise, signs of overt blood loss were absent following evaluation with upper endoscopy and colonoscopy. AIHA was suspected after blood smear and flow cytometry ruled out other hematological disorders, such as nocturnal paroxysmal hemoglobinuria. Further serological workup performed by the American Red Cross was diagnostic for AIHA. A direct antiglobulin test (DAT) was positive for poly‐specific anti‐human globulin (AHG), anti‐IgG, and anti‐C3. Also, her serum tested positive for elevated cold agglutinin titers (1:80). The presence of anti‐C3 and increased cold agglutinin titers fulfills the diagnostic criteria for cAIHA.

Prior to blood transfusion, blood typing and ABO‐Rh cross‐matching were accomplished. Alloantibody screening was negative, so she received 7 units of compatible blood. For immunosuppression, rituximab and prednisone were started. Ferrous sulfate, vitamin B12, and folate were added to promote RBC production. After 3 months, her Hgb recovered to 11.4, but then it began to decrease. The addition of cyclosporine had no effect on her anemia, so the medical team recommended a total splenectomy. She underwent surgery in April 2023, and then she completed six rounds of rituximab and bendamustine. After the operation, her Hgb improved to 8.6. However, it was complicated by postoperative portal vein thrombosis, thus requiring yearlong anticoagulation with Eliquis.

In July 2024, she developed bilateral leg swelling and hepatomegaly. Her symptoms were initially treated with Lasix and then Bumex therapy. Imaging workup was negative for portal hypertension. Due to her elevated ferritin (1220 ng/mL), testing was performed to rule out hemochromatosis. Genetic testing revealed that she carries a copy of the C282Y gene, thus diagnosing her with heterozygous hemochromatosis. Classically, heterozygous patients present with no overt organ dysfunction. Before she could undergo further testing, she developed ascites and weight loss.

**FIGURE 1:**
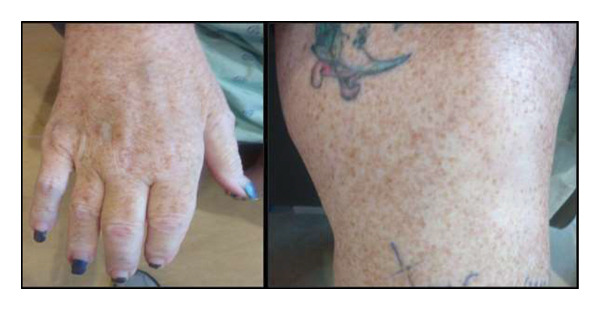
Patient exhibits extensive skin freckling throughout her body. The left image is of her right hand, while the right image is of her right knee.

During her current hospital admission (i.e., September 2024), she presented with a 2‐month history of abdominal distension and 35 lbs of weight loss. Her temperature (98.4°F), blood pressure (154/72 mmHg), heart rate (92 bpm), respiratory rate (18 bpm), and O_2_ saturation (96%) were unremarkable. Lab workup was significant for anemia below baseline (Hgb 7.8, g/dL); her previous Hgb was 9 g/dL. Other lab findings included an elevated creatinine (1.03 mg/dL) and hypoalbuminemia (albumin 1.6 g/dL). A physical exam revealed significant abdominal distension and extensive freckling with a bronze skin tone (Figure 1).

**FIGURE 2:**
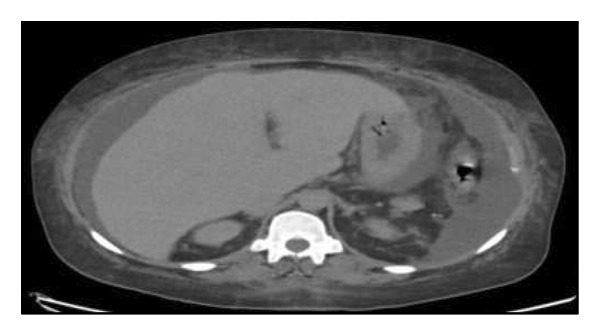
Abdomen and pelvis CT scan, revealing moderate ascites and irregular liver contours.

A CT scan revealed anasarca and moderate ascites with an irregular liver contour, but a normal portal vein (Figure 2). An abdominal ultrasound showed diffuse hepatomegaly that suggested cirrhosis. Although her aspartate transaminase (11 U/L) and alanine transaminase (4 U/L) were normal, her alkaline phosphatase (141 U/L) was elevated. Coagulation studies revealed an elevated prothrombin time (15.3 s) and international normalized ratio (1.3), but a normal activated partial thromboplastin time (32 s). Further hepatic workup revealed an abnormal iron study, which showed decreased iron (29 μg/dL) and total iron binding capacity (115 μg/dL), but increased ferritin (1486 ng/mL). An infectious etiology was ruled out by a negative viral hepatitis panel.

Prophylactic ceftriaxone was administered, and she underwent paracentesis with 4.5 L of fluid removed. The removed fluid was characterized by low albumin (< 1.5 g/dL), reduced lactate dehydrogenase (< 25 U/L), absent malignant cells, and a negative culture. A liver biopsy showed hepatic parenchyma with significant centrilobular sinusoidal dilatation and hepatocyte atrophy. Additional biopsy findings included hemosiderosis and minimal macrovesicular steatosis (< 5%). This histopathology can be seen in venous outflow obstruction and chronic heart failure.

The biopsy was further analyzed by Mayo Clinic Laboratories to determine the extent of liver iron deposition. The studies showed a normal copper concentration (40 mcg/g) and a significantly elevated iron concentration (3499 mcg/g). The hepatic iron index was also high at 1.3 mcmol/g/yr (i.e., normal index is < 1). A hepatic index of 1.0–1.9 can indicate heterozygous hemochromatosis, which supports her previous diagnosis.

Her ascites was due to acute decompensated cirrhosis, most likely secondary to heterozygous hemochromatosis complicated by cAIHA. Hemolysis from cAIHA was assessed by monitoring daily Hgb levels, which improved to 8.4 g/dL. After a 5‐day hospital course, her ascites significantly improved, and she was able to be discharged (Table 1). Ferrous sulfate was discontinued from the medication list due to her high liver iron concentration. Also, her Bumex dose was adjusted to better control her volume status.

**TABLE 1:** A timeline of the major events surrounding our patient’s cAIHA and heterozygous hemochromatosis diagnosis, and her treatment for acute decompensated cirrhosis.

Clinical timeline of patient diagnosis and treatment	
January 2023	A diagnosis of cAIHA is made. She begins rituximab and prednisone
April 2023	Her anemia does not improve with medical therapy. She undergoes splenectomy followed by six rounds of rituximab and bendamustine. Afterward, her anemia improves
July 2024	She develops bilateral lower extremity edema and hepatomegaly. Her edema is treated with Lasix and then Bumex. An imaging workup was negative for portal hypertension
August 2024	Genetic testing confirms a diagnosis of heterozygous hemochromatosis
September 2024	She is admitted for new onset ascites and weight loss. Imaging is suspicious for cirrhosis, and a liver biopsy confirms iron overload. She improves with paracentesis and a Bumex dose adjustment

## 3. Discussion

Hemochromatosis and cAIHA are two rare hematologic conditions that affect the body very differently, thus complicating diagnosis and treatment. This case report discusses the evaluation of a patient with acute decompensated cirrhosis secondary to heterozygous hemochromatosis complicated by cAIHA.

Hemochromatosis is a genetic disorder characterized by increased iron absorption leading to excessive iron accumulation in tissue (e.g., liver, pancreas, and skin) [13, 14]. The condition has a diverse range of presentations; common examples include diabetes, cirrhosis, skin discoloration, and hypogonadism. Our patient carries a single copy of the C282Y gene, thus making her positive for heterozygous hemochromatosis. Heterozygous patients are classically asymptomatic; however, they do exhibit increased iron absorption. Treatment is achieved with phlebotomy, chelation, and lifestyle changes [15]. Lifestyle changes can include avoiding iron (e.g., supplements and fortified foods), limiting alcohol consumption, and reducing vitamin C intake. For our patient, it is important to note that phlebotomy would worsen her underlying anemia.

Treatment for cAIHA is multifactorial and depends on symptom severity. Monoclonal antibodies (e.g., rituximab) prevent the immune system from producing autoantibodies that bind RBCs. Blood transfusions are reserved for unstable patients; however, they should be administered cautiously because autoantibodies can hemolyze transfused blood [16]. Blood transfusions should also be warmed, so that it does not provoke hemolysis. Lifestyle changes, such as avoiding cold temperatures, can be taken to decrease autoantibody activity. For severe cAIHA that is refractory to medical treatment, splenectomy is often pursued as a third‐line treatment [17]. Although not regularly recommended, splenectomy has been shown to improve anemia in patients with severe AIHA, as seen with our patient [18].

The development of acute decompensated cirrhosis most likely resulted from her heterozygous hemochromatosis. Although usually asymptomatic, heterozygous patients still exhibit increased iron absorption. In the background of cAIHA, chronic hemolysis leads to increased serum iron that is available for tissue deposition. Also, she was taking regular iron supplements to promote RBC production. Taken together, her increased iron exposure resulted in symptomatic heterozygous hemochromatosis, as evidenced by her cirrhosis symptoms. Once it was shown that her hepatic iron levels were significantly elevated, it became clear that iron supplementation must be discontinued. Blood transfusions were avoided to prevent excess iron exposure. Also, supportive strategies (e.g., maintaining a warm environment) were employed to limit hemolysis and improve her Hgb. Treatment of her cirrhosis with paracentesis and Bumex alleviated her other symptoms.

## 4. Conclusion

The case report represents a rare instance of symptomatic heterozygous hemochromatosis due to underlying cAIHA. In patients with an atypical presentation, a thorough investigation must be performed to evaluate for contributing hematological conditions. In our patient with cirrhosis not due to viral hepatitis, alcohol, or fatty liver disease, a careful examination of her underlying hematologic conditions revealed the ultimate cause of her symptoms. By understanding the complementary pathophysiology between cAIHA and heterozygous hemochromatosis, we were able to develop an effective treatment plan that reduces her risk of further iron overload.

## Author Contributions

J.H.F., P.K.J., and S.J. conducted the literature review and wrote the manuscript. F.S. and A.G. contributed to the patient’s clinical management. A.G. and J.H.F. edited the manuscript.

## Funding

The authors have nothing to report.

## Disclosure

All authors have read and approved the final manuscript for submission.

## Consent

The patient offered written informed consent for the publication of this case report. Consent was provided using a standardized institutional consent form.

## Conflicts of Interest

The authors declare no conflicts of interest.

## Data Availability

The authors have nothing to report.

## References

[1] Klein N. P. , Ray P. , Carpenter D. et al., Rates of Autoimmune Diseases in Kaiser Permanente for Use in Vaccine Adverse Event Safety Studies, Vaccine. (2010) 28, no. 4, 1062–1068, 10.1016/j.vaccine.2009.10.115, 2-s2.0-73949159847. 19896453

[2] Maquet J. , Lafaurie M. , Walter O. et al., Epidemiology of Autoimmune Hemolytic Anemia: A Nationwide Population-based Study in France, American Journal of Hematology. (2021) 96, no. 8, E291–E293, 10.1002/ajh.26213. 33930203

[3] Mulder F. V. M. , Evers D. , de Haas M. et al., Severe Autoimmune Hemolytic Anemia; Epidemiology, Clinical Management, Outcomes and Knowledge Gaps, Frontiers in Immunology. (2023) 14, 10.3389/fimmu.2023.1228142. PMC10545865 37795092

[4] Berentsen S. , New Insights in the Pathogenesis and Therapy of Cold Agglutinin-Mediated Autoimmune Hemolytic Anemia, Frontiers in Immunology. (2020) 11, 10.3389/fimmu.2020.00590. PMC7154122 32318071

[5] Loriamini M. , Cserti-Gazdewich C. , and Branch D. R. , Autoimmune Hemolytic Anemias: Classifications, Pathophysiology, Diagnoses and Management, International Journal of Molecular Sciences. (2024) 25, no. 8, 10.3390/ijms25084296. PMC11049952 38673882

[6] Despotovic J. M. and Kim T. O. , Cold AIHA and the Best Treatment Strategies, Hematology, ASH Education Program. (2022) 2022, no. 1, 90–95, 10.1182/hematology.2022000369. PMC9821124 36485161

[7] Jager U. , Barcellini W. , Broome C. M. et al., Diagnosis and Treatment of Autoimmune Hemolytic Anemia in Adults: Recommendations from the First International Consensus Meeting, Blood Reviews. (2020) 41, 10.1016/j.blre.2019.100648. 31839434

[8] Gabbard A. P. and Booth G. S. , Cold Agglutinin Disease, Clinical Hematology International. (2020) 2, no. 3, 95–100, 10.2991/chi.k.200706.001. 34595449 PMC8432332

[9] Berentsen S. , Barcellini W. , D′Sa S. et al., Cold Agglutinin Disease Revisited: A Multinational, Observational Study of 232 Patients, Blood. (2020) 136, no. 4, 480–488, 10.1182/blood.2020005674. 32374875

[10] Badireddy M. and Baradhi K. M. , Chronic Anemia, StatPearls, 2024, Treasure Island (FL).

[11] Hansen D. L. , Moller S. , and Frederiksen H. , Survival in Autoimmune Hemolytic Anemia Remains Poor, Results from a Nationwide Cohort With 37 Years of Follow-Up, European Journal of Haematology. (2022) 109, no. 1, 10–20, 10.1111/ejh.13764. 35276014 PMC9314695

[12] Zanella A. and Barcellini W. , Treatment of Autoimmune Hemolytic Anemias, Haematologica. (2014) 99, no. 10, 1547–1554, 10.3324/haematol.2014.114561, 2-s2.0-84907563024. 25271314 PMC4181250

[13] Kane S. F. , Roberts C. , and Paulus R. , Hereditary Hemochromatosis: Rapid Evidence Review, American Family Physician. (2021) 104, no. 3, 263–270. 34523883

[14] Kowdley K. V. , Brown K. E. , Ahn J. , and Sundaram V. , ACG Clinical Guideline: Hereditary Hemochromatosis, American Journal of Gastroenterology. (2019) 114, no. 8, 1202–1218, 10.14309/ajg.0000000000000315, 2-s2.0-85071066974. 31335359

[15] Porter J. L. and Rawla P. H. , Hemochromatosis, StatPearls, 2024, Treasure Island (FL).

[16] Su J. , Punekar R. , Morales Arias J. , and Jain N. , Cold Agglutinin Disease Transfusion Practices in the United States: An Electronic Medical Record-Based Analysis, Blood. (2019) 134, no. Supplement_1, 10.1182/blood-2019-122209.

[17] Cvetkovic Z. , Pantic N. , Cvetkovic M. et al., The Role of the Spleen and the Place of Splenectomy in Autoimmune Hemolytic Anemia-A Review of Current Knowledge, Diagnostics. (2023) 13, 10.3390/diagnostics13182891. PMC10527817 37761258

[18] Okamoto S. , Urade T. , Yakushijin K. et al., Successful Management of Refractory Autoimmune Hemolytic Anemia with Cold Agglutinin Disease with Splenectomy: A Case Report With Review of Literature, Kobe Journal of Medical Sciences. (2023) 68, no. 1, E30–E34. 36647084 PMC10117625

